# Supplementary material for: Evaluation of β-Lactamase Enzyme Activity in Outer Membrane Vesicles (OMVs) Isolated from Extended Spectrum β-Lactamase (ESBL) Salmonella Infantis Strains
Source: Antibiotics (Basel). 2023 Apr 13;12(4):744. doi: 10.3390/antibiotics12040744 (PMC10135247; doi:10.3390/antibiotics12040744)
Supplement: Supplementary file 1 [file antibiotics-12-00744-s001.zip › antibiotics-2266086-supplementary.pdf]

TABLE S1. β-lactamase enzymes values (mU/mg) in OMVs concentrate, eluted and 0.45 μm filtrate

	**β-lactamase activity (mU/mg)**		
Strains	OMVs concentrate	Eluted	Filtrate 0.45 μm
1	355,45 ± 156,06	346,30 ± 33,71	175,77 ± 156,06
2	372,48 ± 6,00	118,70 ± 38,77	121,51 ± 6,00
3	507,88 ± 98,04	346,30 ± 139,38	365,66 ± 98,04
4	336,47 ± 145,94	297,69 ± 306,67	242,61 ± 145,94
5	1359,26 ± 167,39	289,34 ± 163,52	263,59 ± 167,39
